# Supplementary material for: A Randomized Controlled Trial of Cognitive Behavioral Therapy for Insomnia During Early Recovery from Alcohol Use Disorder Among Veterans
Source: medRxiv. 2025 Jan 5:2025.01.03.25319973. Preprint. [Version 1] doi: 10.1101/2025.01.03.25319973 (PMC11722466; doi:10.1101/2025.01.03.25319973)
Supplement: Supplement 1 [file NIHPP2025.01.03.25319973v1-supplement-1.pdf]

medRxiv preprint doi: https://doi.org/10.1101/2025.01.03.25319973; this version posted January 5, 2025. The copyright holder for this preprint (which was not certified by peer review) is the author/funder, who has granted medRxiv a license to display the preprint in perpetuity.
This article is a US Government work. It is not subject to copyright under 17 USC 105 and is also made available for use under a CC0 license.

Supplementary Table Captions

Supplementary Table S1. Treatment credibility and fidelity scores (mean ± standard deviation) across treatment groups

Supplementary Table S2. Differences in baseline demographic and clinical variables across the groups based on insomnia treatment response at the end of week 8 of treatment

Supplementary Table S3. Adverse events with treatment

Supplementary Figure Captions

Supplementary Figure S1. CONSORT flow chart for flow of subjects in the study

Supplementary Figure S2. Percent days abstinent (PDA) in the groups based on insomnia treatment outcomes.

medRxiv preprint doi: https://doi.org/10.1101/2025.01.03.25319973; this version posted January 5, 2025. The copyright holder for this preprint (which was not certified by peer review) is the author/funder, who has granted medRxiv a license to display the preprint in perpetuity.
This article is a US Government work. It is not subject to copyright under 17 USC 105 and is also made available for use under a CC0 license.

Supplementary Table S1. Treatment credibility and fidelity scores (mean ± standard deviation) across groups

**1. QDT Versus CBT-I**			
**Characteristic**	**QDT**	**CBT-I**	**p-value**
Therapist adherence to protocol (%)	93.75 ± 12.40	97.61 ± 3.58	0.89
Patient expectation (at baseline)	34.38 ± 13.16	29.17 ± 10.55	0.11
Treatment Fidelity			
- belief that alcohol would help them sleep better (%)	15.00	14.00	0.31
- change in KAQ total score (with treatment)	0.51 ± 2.34	0.07 ± 1.44	0.41
Treatment Credibility			
- TEQ item 1 (pretreatment)	5.74 ± 1.71	5.34 ± 1.47	0.34
- TEQ item 2 (pretreatment)	5.38 ± 1.80	5.65 ± 1.36	0.52
- TEQ item 3 (pretreatment)	5.54 ± 1.62	5.89 ± 1.44	0.38
- TEQ item 4 (pretreatment)	6.61 ± 1.17	6.26 ± 1.25	0.27
- TEQ item 5 (pretreatment)	5.73 ± 1.46	5.53 ± 1.47	0.61
- Change in TEQ total score with treatment	12.03 ± 17.40	7.96 ± 16.32	0.39
**2. Dropouts versus completers**			
**Characteristic**	**Dropouts**	**Completers**	**p-value**
Patient expectation (at baseline)	36.33 ± 16.84	31.10 ± 11.43	0.48
KAQ total score (baseline)	7.42 ± 2.22	6.56 ± 1.92	0.35
TEQ total score (baseline)	30.28 ± 3.19	27.05 ± 8.66	0.33

Addendum: 1) Patient adherence to treatment. *a. Sleep Restriction.* Twelve subjects showed partial noncompliance, with 75% being noncompliant for one week, especially treatment week 2. *b. Stimulus Control.* No subjects met criteria for noncompliance. QDT = quasi-desensitization therapy; CBT-I = cognitive behavioral therapy for insomnia.

medRxiv preprint doi: https://doi.org/10.1101/2025.01.03.25319973; this version posted January 5, 2025. The copyright holder for this preprint (which was not certified by peer review) is the author/funder, who has granted medRxiv a license to display the preprint in perpetuity. This article is a US Government work. It is not subject to copyright under 17 USC 105 and is also made available for use under a CC0 license.

Supplementary Table S2. Differences in baseline demographic and clinical variables across the groups based on insomnia treatment response at the end of week 8 of treatment

Domain	Variable	Remission (N=24)	Response (N=12)	Non-response (N=20)	p-value
Demographic	Age	52.9±10.1	52.7±8.9	51.8±8.2	0.76
	Gender (males)	22 (22/24)	10 (10/12)	19 (19/20)	0.60
	Marital status (single)	20 (20/24)	11 (11/12)	19 (19/20)	0.55
Sleep	ISI total score	18.5±4.2	21.3±3.0	18.8±2.8	***0.07***
	SOL	47.3±33.2	61.8±55.0	49.0±36.7	0.89
	WASO	39.0±33.1	28.5±23.6	38.3±27.7	0.63
	TST	332.7±109.0	347.6±81.5	327.3±91.9	0.85
Alcohol	Days abstinent before Tr.	120.5±97.8	112.1±117.9	117.0±121.8	0.98
	PDA	33.3±33.7	36.7±29.4	27.8±34.9	0.74
	Drinks per day	9.4±8.1	10.3±10.3	8.0±7.8	0.66
	Percent HDD	60.7±37.8	51.6±38.7	51.5±42.6	0.69
	PACS total score	7.7±6.7	11.3±8.5	9.3±6.6	0.37
Psychiatric	BDI total score	12.6±9.8	26.1±11.2	19.6±10.1	**0.001**
	STAI-trait score	46.8±4.7	47.3±7.2	43.2±6.1	0.08
	PTSD status (positive)	8 (8/24)	8 (8/12)	11 (11/20)	0.14
	Psychotropic meds	9 (16.07%)	11 (19.64%)	15 (26.79%)	**0.002**
Medical	Charlson's Index	1.8±1.5	2.1±1.6	2.2±2.0	0.74

Legend: *Remission subgroup* (N = 24) included subjects with an ISI total score <8 at week 8; *Response subgroup* (N = 12) included treatments responders, i.e., those with a per-post change in ISI<7; *Non-response subgroup* included those with neither response nor remission; ISI = Insomnia Severity Index; SOL = Sleep Onset Latency; WASO = Wake After Sleep Onset time; TST = Total Sleep Time; Dur. Of abstinence = duration of abstinence from alcohol before onset of behavioral treatment for insomnia; PDA = Percent Days Abstinent; Percent HDD – Percent Heavy Drinking Days; PACS = Penn Alcohol Craving Scale total score; BDI total score = Beck Depression Inventory total score excluding the sleep item; STAI-trait score = State Trait Anxiety Inventory – Trait subscale score; Post-hoc testing for the BDI scores using Bonferroni's test shows remitters < responders (p = 0.001) but remitters < non-responders (p = 0.08).

medRxiv preprint doi: https://doi.org/10.1101/2025.01.03.25319973; this version posted January 5, 2025. The copyright holder for this preprint (which was not certified by peer review) is the author/funder, who has granted medRxiv a license to display the preprint in perpetuity.
This article is a US Government work. It is not subject to copyright under 17 USC 105 and is also made available for use under a CC0 license.

Supplementary Table S3. Adverse events with treatment

Event	QDT (N = 32)	CBT-I (N = 31)	p-value
Abdominal pain	0 (0.00%)	1 (1.59%)	0.49
Cerebrovascular accident	1 (1.59%)	0 (0.00 %)	1.00
Alcohol detoxification	2 (3.17%)	4 (6.35%)	0.42
Residential treatment for drinking	0 (0.00%)	1 (1.59%)	0.49
Pneumonia	0 (0.00%)	1 (1.59%)	0.49
Total	3 (4.76)	7 (11.11%)	0.18

Legend: CBT-I = Cognitive Behavioral Therapy for Insomnia; QDT = Quasi-Desensitization Therapy

medRxiv preprint doi: https://doi.org/10.1101/2025.01.03.25319973; this version posted January 5, 2025. The copyright holder for this preprint (which was not certified by peer review) is the author/funder, who has granted medRxiv a license to display the preprint in perpetuity.
This article is a US Government work. It is not subject to copyright under 17 USC 105 and is also made available for use under a CC0 license.

Supplementary Figure S1. CONSORT flow chart for flow of subjects in the study

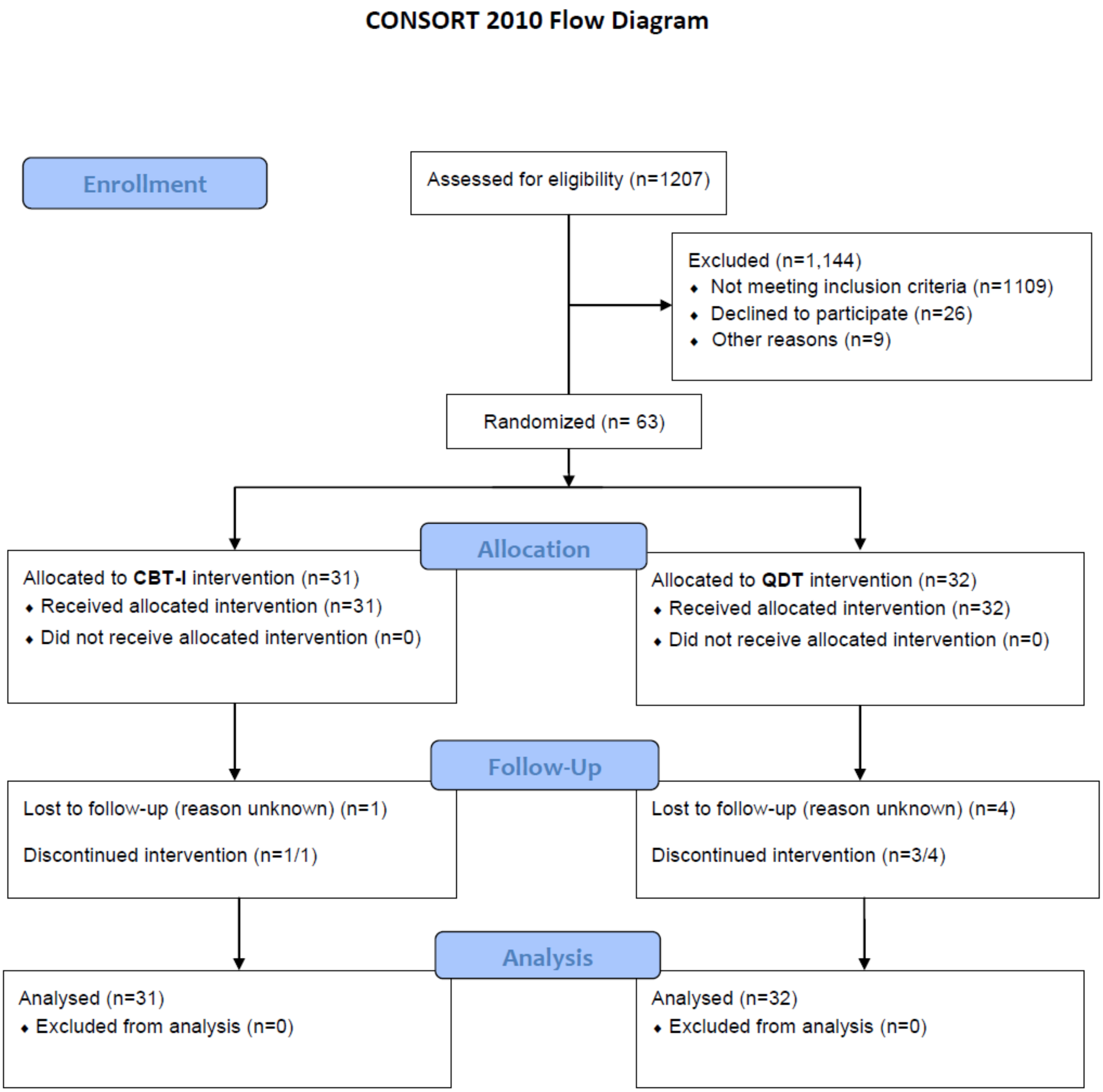

medRxiv preprint doi: https://doi.org/10.1101/2025.01.03.25319973; this version posted January 5, 2025. The copyright holder for this preprint (which was not certified by peer review) is the author/funder, who has granted medRxiv a license to display the preprint in perpetuity.
This article is a US Government work. It is not subject to copyright under 17 USC 105 and is also made available for use under a CC0 license.

Supplementary Figure S2. Percent days abstinent (PDA) in the groups based on insomnia treatment outcomes.

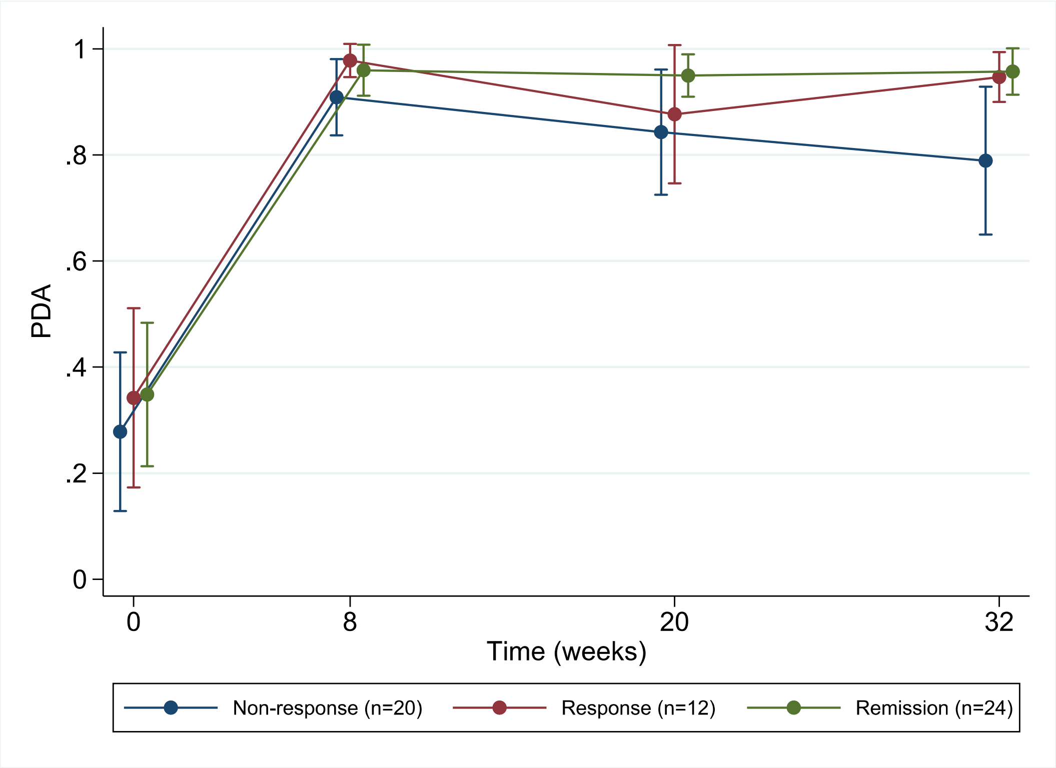

Legend: *Non-response subgroup* (N = 20) included those with neither response nor remission; *Response subgroup* (N = 12) included treatments responders, i.e., those with a per-post change in ISI<7; *remission subgroup* (N = 24) included subjects with an ISI total score <8 at week 8. Subjects with missing data who were excluded (N = 7). The results showed that there was no difference in the PDA between the subgroups over time (p=0.65).
